# *Plasmodium* heme biosynthesis: To be or not to be essential?

**DOI:** 10.1371/journal.ppat.1006511

**Published:** 2017-09-28

**Authors:** Daniel E. Goldberg, Paul A. Sigala

**Affiliations:** 1 Departments of Medicine and Molecular Microbiology, Washington University School of Medicine, St. Louis, Missouri, United States of America; 2 Department of Biochemistry, University of Utah School of Medicine, Salt Lake City, Utah, United States of America; University of Wisconsin Medical School, UNITED STATES

Sequencing of the *Plasmodium falciparum* genome in 2002 sparked hopes that metabolic pathway annotation could identify new parasite-specific targets for therapeutic intervention [1]. Cashing in on this potential has proved challenging because many pathways have unexpected properties and/or are only required in certain host environments. Heme biosynthesis has been considered as a possible antimalarial target. Our understanding of heme acquisition mechanisms and the role of heme biosynthesis in different stages of *Plasmodium* parasite development has evolved considerably in recent years. In this Pearl, we summarize recent major findings and highlight remaining questions.

## *Plasmodium* parasites require heme as a metabolic cofactor

**Fig 1:**
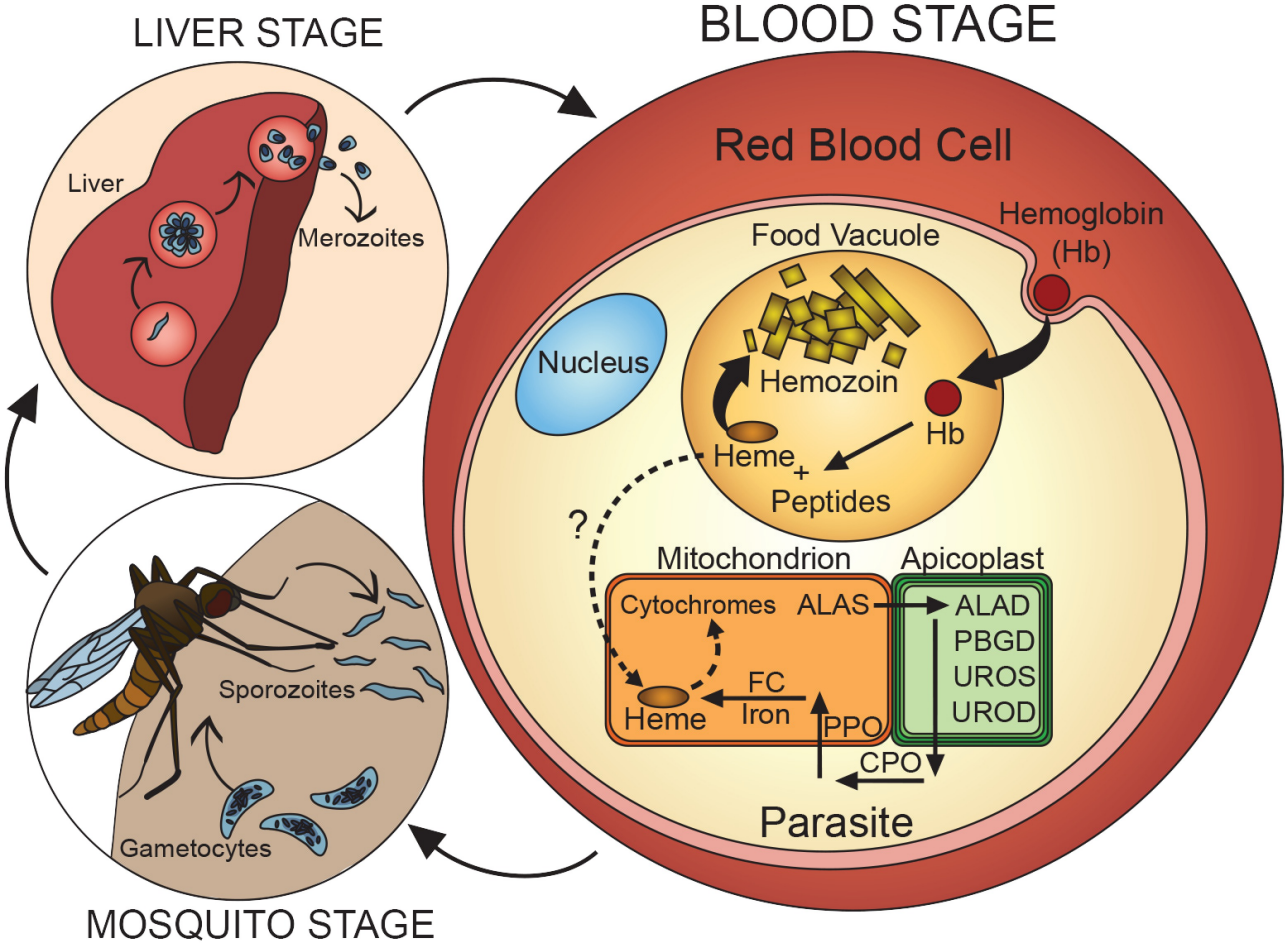
*Plasmodium* parasites require heme as a metabolic cofactor throughout their life cycle. *Plasmodium* enzyme abbreviations are consistent with those used at the *Plasmodium* Genomics Resource (PlasmoDB.org). Abbreviations: ALAD, aminolevulinic acid dehydratase; ALAS, aminolevulinic acid synthase; CPO, coproporphyrinogen oxidase; FC, ferrochelatase; PBGD, porphobilinogen deaminase; PPO, protoporphyrinogen oxidase; UROD, uroporphyrinogen decarboxylase; UROS, uroporphyrinogen synthase.

Eukaryotic malaria parasites have retained a mitochondrion and the heme-dependent cytochrome components of the electron transport chain (Fig 1), including cytochromes b and c_1_ of Complex III, soluble cytochrome c, and the CoxI subunit of Complex IV [2]. The antimalarial drug atovaquone kills parasites in blood, mosquito, and liver stages by binding to cytochrome b, where it inhibits ubiquinone association and blocks electron transport to Complex III, underscoring the critical role of heme-dependent electron transport throughout the parasite life cycle [3, 4]. Prior work has suggested that blood-stage *Plasmodium* parasites may only require the electron transport chain for oxidative recycling of ubiquinone to support the essential mitochondrial enzyme dihydroorotate dehydrogenase (DHOD) because episomal expression of ubiquinone-independent yeast DHOD rescues atovaquone lethality [5]. Mounting evidence strongly suggests that parasites also require heme-dependent electron transport for ATP synthesis via mitochondrial oxidative phosphorylation during mosquito-stage growth [6, 7]. It remains a future challenge to assess whether parasites require heme to support essential cellular processes other than cytochrome-mediated electron transport.

## Parasites do not require heme biosynthesis for blood-stage growth

**Fig 2:**
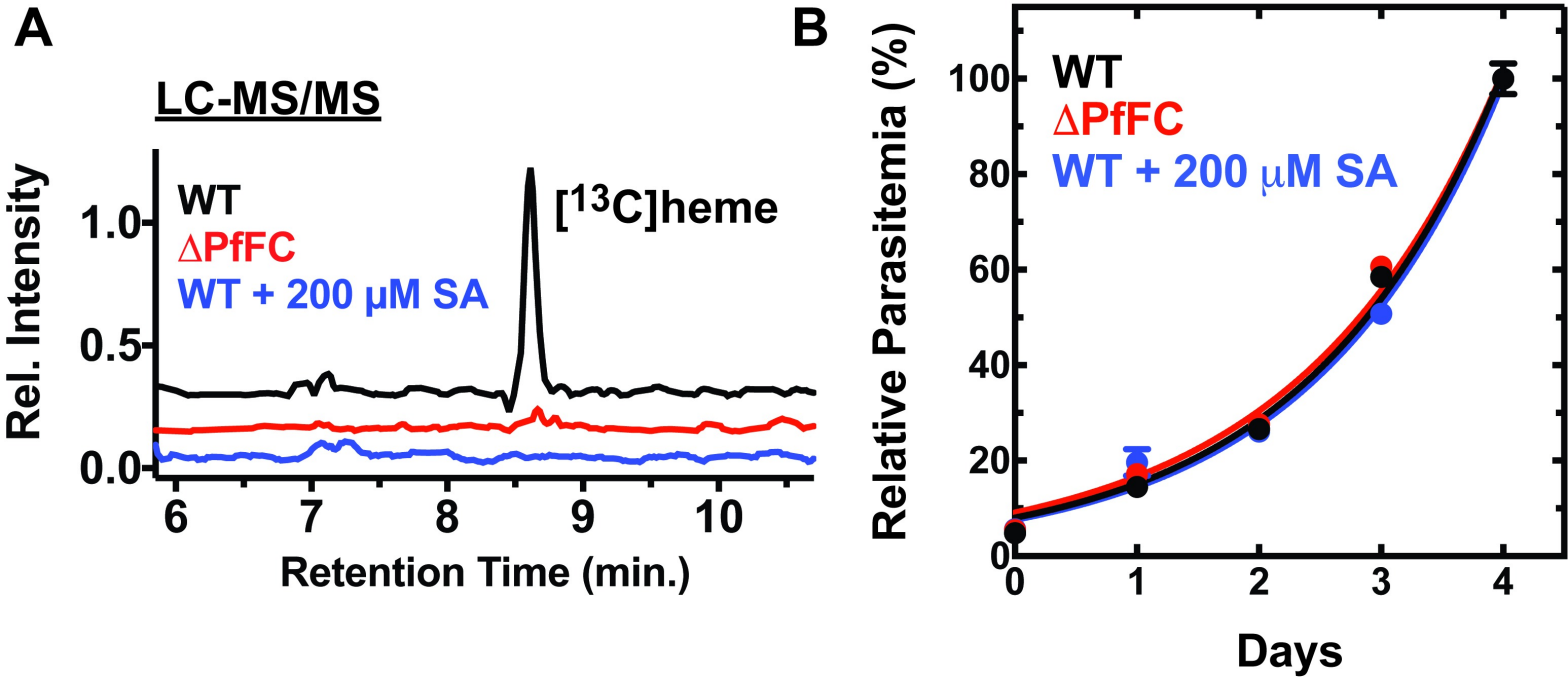
Ablating heme biosynthesis has no effect on blood-stage *P*. *falciparum* growth. (A) Mass spectra (from [12]) for detection of [^13^C]heme biosynthesized from ^13^C_4_-ALA in WT *P*. *falciparum* D10 parasites, ΔFC parasites, or WT parasites grown in 200 μM SA. Spectra were normalized to the intensity of the heme peak in the WT parasite sample and offset to avoid baseline overlap. (B) Growth of asynchronous WT or ΔFC D10 parasites in the absence or presence of 200 μM SA (from [13]). Measured parasitemia values for each parasite line were normalized to the respective parasitemia on day 4 and fit with an exponential growth equation. Abbreviations: ΔPfFC, genetic knockout of P. falciparum ferrochelatase; SA, succinylacetone; WT, wild-type.

How do parasites obtain heme to support mitochondrial cytochrome function? During blood-stage growth, *Plasmodium* parasites develop within the most heme-rich cell in the human body and liberate vast amounts of host-derived heme during hemoglobin digestion within the acidic food vacuole [8]. Despite access to this abundant heme source, intraerythrocytic parasites encode and express all enzymes for a complete heme biosynthesis pathway (Fig 1) [9]. This pathway was originally proposed to be essential and therefore a possible therapeutic target based on observations that succinylacetone inhibits heme biosynthesis and kills parasites at millimolar concentrations [10, 11]. However, subsequent studies of *Plasmodium berghei* and *P*. *falciparum* reported the successful gene deletion of ferrochelatase (FC), the last enzyme in the parasite pathway. These ΔFC parasites (where Δ indicates a disrupted gene) grew indistinguishably from wild-type (WT) parasites [11, 12] and developed sexually to stage V gametocytes, despite a failure to synthesize heme from the ^13^C-labeled precursor 5-aminolevulinic acid (ALA) (Fig 2) [12]. Furthermore, much lower concentrations of succinylacetone blocked de novo heme synthesis but had no effect on parasite growth (Fig 2) [11–13], strongly suggesting that parasite death from succinylacetone at high concentrations was due to off-target toxicity. Additional studies have reported the disruption of 4 other heme biosynthesis genes (ALA synthase [ALAS], porphobilinogen deaminase, uroporphyrinogen decarboxylase, and coproporphyrinogen oxidase), all without a detectable effect on blood-stage parasite growth [11–14]. These studies strongly support the conclusion that parasites do not depend on heme biosynthesis during blood-stage infection. Parasites must therefore have mechanisms to scavenge host-derived heme to satisfy cofactor requirements for mitochondrial cytochromes, and it remains an exciting future challenge to identify and understand these mechanisms. Labile heme levels in the parasite cytoplasm have been estimated to be about 1 μM [15]. This concentration increases with chloroquine treatment [15, 16], which blocks heme sequestration in the food vacuole [8], suggesting that this compartment may be the dominant source of cytoplasmic heme. Preliminary studies suggest that host heme is imported into the parasite mitochondrion [11], but much more work is needed to dissect and understand heme trafficking pathways in parasites.

Recent studies reported that *P*. *falciparum* parasites showed reduced growth in blood from patients with congenital deficiencies in human ferrochelatase (FECH) or in blood from normal donors treated with the FECH inhibitor N-methylprotoporphyrin (NMPP) [17, 18]. From these observations, the authors proposed that blood-stage parasites do require heme biosynthesis by a pathway involving human FECH imported from the host erythrocyte into parasites. This proposal is difficult to reconcile with the prior observations that all heme biosynthesis can be ablated in parasite-infected red blood cells without a corresponding reduction in parasite growth (Fig 2) [11, 12]. In addition, *P*. *falciparum* parasites predominantly invade mature erythrocytes, which lose mitochondria during the terminal differentiation of precursor reticulocytes and thus lack the enzymes targeted to this organelle, including human FECH [19–21]. Slowed parasite growth in blood from congenitally FECH-deficient patients may reflect the effects of developmental defects in these cells, incurred during earlier stages of erythropoiesis. The deleterious growth effects of exogenous NMPP may therefore arise from off-target drug toxicity (with rescue by protoporphyrin IX [PPIX], explained by competition for the same off-target or uptake receptor) [17].

### Parasite heme biosynthesis is essential for mosquito and liver stage infections

In contrast to their indistinguishable blood-stage growth relative to WT parasites, the ΔALAS and ΔFC *P*. *berghei* and *P*. *falciparum* parasites displayed a complete developmental arrest during mosquito-stage growth. Although different studies have observed contrasting developmental failures before [11, 12] or after [14, 22] oocyst formation, in all cases, the knockout parasites failed to form mature salivary gland sporozoites such that bite-back experiments of naïve mice by mutant-infected mosquitoes failed to produce blood-stage patency in these animals [11, 14, 22]. The mosquito-stage growth defect of ΔALAS *P*. *berghei* parasites could be chemically rescued by including 5-ALA, the product of ALAS catalysis, in the mosquito feeding solution in order to enable maturation to salivary gland sporozoites [11, 14]. These studies uniformly support the conclusion that *Plasmodium* parasites require heme biosynthesis during growth within mosquitoes. This differing dependence relative to blood stages may reflect both the lower availability of host heme within mosquitoes and/or an enhanced parasite reliance on heme-dependent mitochondrial electron transport for ATP synthesis [6, 7].

To test parasite reliance on heme biosynthesis during liver-stage growth, 2 studies with *P*. *berghei* allowed mosquitoes infected with ALA-rescued ΔALAS parasites to feed on naïve mice and then evaluated whether parasites could complete liver-stage development and establish subsequent blood-stage infection. One study failed to detect blood-stage patency [11], suggesting an essential role for heme biosynthesis during parasite infection of liver hepatocytes. However, the other group reported a reduction in liver-stage ΔALAS parasite load but an otherwise normal ability of the knockout parasites to complete liver-stage development and establish blood-stage infection [14]. These conflicting observations, for which both groups propose explanations [14, 23], have prevented a strong conclusion. Host hepatocytes actively synthesize heme via their own pathway, which includes 4 cytoplasmic enzymes. The heme intermediates produced by these cytosolic host enzymes could, in principle, be scavenged by invading parasites to bypass the ΔALAS mutation and thus complicate the interpretation of this mutant. A recent study of ΔFC *P*. *berghei* parasites [22] has definitively answered this question because this mutation cannot be bypassed by scavenging upstream intermediates. Fluorescently tagged ΔFC parasites were bridged past their mosquito-stage defect by crossing them to WT parasites in order to produce heterozygous polyploid oocysts with both disrupted and WT FC alleles. Haploid ΔFC sporozoites resulting from the polyploid oocysts infected liver hepatocytes in vitro and in mouse bite-back experiments but failed to form liver merosomes or establish blood-stage patency [22]. These observations strongly support the conclusion that heme biosynthesis is critical for liver-stage parasite development.

## Is heme biosynthesis an antimalarial drug target?

Because blood-stage parasites do not depend on de novo heme synthesis for growth and viability, drugs that inhibit heme biosynthesis enzymes are unlikely to provide effective antimalarial therapies. However, if such inhibitors were taken up with gametocytes by blood-feeding mosquitoes and persisted at high concentrations within the insect, then the stable inhibition of parasite enzymes could, in principle, prevent further development into sporozoites and thus block transmission to new human hosts. Because heme biosynthesis is essential for liver-stage development, the inhibition of parasite enzymes could provide effective prophylaxis against subsequent blood-stage malaria. However, such drugs would need to specifically target parasite enzymes and not inhibit the essential human enzymes.

As an alternative to therapeutic inhibition, the stimulation of porphyrin biosynthesis by exogenous ALA has recently been shown to effectively kill blood-stage parasites via photodynamic interaction with light [13, 24] or synergy with ferrous iron [25]. Exogenous ALA causes intraerythrocytic accumulation of the photoactive heme intermediate PPIX, largely by stimulating the activity of vestigial host enzymes remaining in the erythrocyte cytoplasm [13]. Light, which can be supplied externally or generated in situ within cells via nontoxic chemiluminescent reagents, excites PPIX to produce cytotoxic reactive oxygen species that kill parasites. Methods involving ALA stimulation of PPIX production thus depend predominantly on host cell enzymes outside of the genetic control of parasites, which may limit resistance development. Future work will delineate whether these approaches can provide effective antiparasitic therapy in vivo.

## Frontier questions

Heme is an ancient biological cofactor required by nearly all organisms for cellular viability. By virtue of their intraerythrocytic lifestyle, *Plasmodium* parasites interact with heme as both a toxic by-product of large-scale hemoglobin catabolism and as an essential cofactor for their own metabolism. Understanding how parasites acquire and utilize heme will uncover fundamental adaptations evolved by parasites to survive within different host environments, and this understanding can be harnessed to develop new strategies to target this virulent pathogen. Below, we lay out key unresolved questions at the frontier of our understanding of heme acquisition and utilization by parasites.

1. How active is the parasite heme biosynthesis pathway during intraerythrocytic growth, and do endogenous Gly + succinyl CoA or exogenous serum ALA serve as the dominant precursor? What portion of mitochondrial heme is derived from synthesis versus scavenging?
2. If the parasite heme biosynthesis pathway is not required for blood-stage growth, why are pathway enzymes expressed in this stage?
3. What are the mechanisms involved in heme scavenging and trafficking in parasites?
4. What is the full extent of heme utilization by parasites? Is heme utilization unusually narrow and limited to mitochondrial cytochromes, or is there a broader landscape of noncanonical heme proteins lurking in the unannotated “dark matter” of the genome?
